# Tension Experience Induced By Nested Structures In Music

**DOI:** 10.3389/fnhum.2020.00210

**Published:** 2020-06-24

**Authors:** Lijun Sun, Chen Feng, Yufang Yang

**Affiliations:** ^1^Key Laboratory of Behavioral Science, Institute of Psychology, Chinese Academy of Sciences, Beijing, China; ^2^Department of Psychology, University of Chinese Academy of Sciences, Beijing, China

**Keywords:** tension, resolution, nested structure, LPC, integration

## Abstract

Tension experience is the basis for music emotion. In music, discrete elements are always organized into complex nested structures to convey emotion. However, the processing of music tension in the nested structure remains unknown. The present study investigated the tension experience induced by the nested structure and the underlying neural mechanisms, using a continuous tension rating task and electroencephalography (EEG) at the same time. Thirty musicians listened to music chorale sequences with non-nested, singly nested and doubly nested structures and were required to rate their real-time tension experience. Behavioral data indicated that the tension experience induced by the nested structure had more fluctuations than the non-nested structure, and the difference was mainly exhibited in the process of tension induction rather than tension resolution. However, the EEG data showed that larger late positive components (LPCs) were elicited by the ending chords in the nested structure compared with the non-nested structure, reflecting the difference in cognitive integration for long-distance structural dependence. The discrepancy between resolution experience and neural responses revealed the non-parallel relations between emotion and cognition. Furthermore, the LPC elicited by the doubly nested structure showed a smaller scalp distribution than the singly nested structure, indicating the more difficult processing of the doubly nested structure. These findings revealed the dynamic tension experience induced by the nested structure and the influence of nested type, shedding new light on the relationship between structure and tension in music.

## Introduction

Musical tension is one of the core principles evoking musical emotions, playing an important role in musical listening (Lehne et al., 2013; Lehne and Koelsch, 2015). As the link between auditory stimuli and subjective experience, tension experience relies on the cognition of complex structures through the process of expectation build-up, violation, and fulfillment (Margulis, 2005; Huron, 2006; Rohrmeier and Koelsch, 2012). Indeed the relationship between tension and structure was depicted in the generative theory of tonal music (GTTM; Lerdahl and Jackendoff, 1983) and the tonal tension model (TTM; Lerdahl and Krumhansl, 2007). It was suggested that tension experience was highly hierarchical, based on the harmonic stability of each chord/note in the passage of tonal music. Depending on tonally hierarchical positions in Western tonal music, the patterns of tension and resolution were presented through a tree notation. Through manipulating the tonal function of certain chords, previous studies have corroborated the GTTM and the TTM finding that unstable chords and structural breaches induced tension experience in short chord sequences (Bigand et al., 1996; Bigand and Parncutt, 1999; Steinbeis et al., 2006; Lerdahl and Krumhansl, 2007). However, there are far more complicated structures in real music and structure–tension relationships in music listening.

Discrete elements in music are organized into complex structure through finite state grammar (FSG) and phrase structure grammar (PSG; Rohrmeier et al., 2014; Ma et al., 2018b). It is the PSG, rather than the FSG, that organizes a set of finite elements into infinite sentences/phrases in the form of nested tree structures to express complicated and rich meaning (Chomsky, 1957, 1965; Fitch and Martins, 2014), constituting the core cognitive faculty of the human beings (Fitch and Hauser, 2004; Makuuchi et al., 2009; Dehaene et al., 2015). In terms of PSG, the discrete elements in music are always organized in a subordinate or dominant way (Lerdahl and Jackendoff, 1983; Longuet-Higgins, 1987; Rohrmeier, 2011; Prince and Schmuckler, 2014), such as the harmonic progression of A (the original key)—B (new key)—A (return to the original key), with a new key embedded in the original key at a higher level. Given the importance of PSG in music, it is essential for us to uncover the tension experience induced by the nested structure, which would shed new light on the relationship between structure and emotion in music.

As a core principle in the tension models (Lerdahl and Jackendoff, 1983; Lerdahl and Krumhansl, 2007), prolongational reduction assigns to pitches a hierarchical structure that expresses tension and relaxation. Thus, the type of hierarchical structures in music can influence the way of prolongational reduction and the tension–resolution pattern. Local and simple tension–resolution patterns are organized in a hierarchical fashion, forming a global and complex tension–resolution pattern. Indeed numerous tension arches are usually interweaved into large-scale tension arches in Western music (Koelsch, 2013). For example, in the case of the harmonic progression of C major—F major—C major, the occurrence of the tonal modulation of F major key induces tension experience because out-of-key chords violate the mental representation based on the original tonal context (Steinbeis et al., 2006; Lerdahl and Krumhansl, 2007). Meanwhile, listeners remember the beginning C major key and expect the subsequent unfolding musical events to modulate to the beginning tonality (Meyer, 1956). Thus, when the C major key returns, the listeners would integrate the nested F major key into the C major key context based on their knowledge of nested structure and acquire a resolution experience (Schenker, 1979; Krumhansl and Kessler, 1982). However, the tension experience induced by the nested structure remains unknown.

Although little research has revealed tension–resolution patterns induced by nested structure, the cognitive processing of nested structure and long-distance dependence in music has been explored by several studies. Behavioral studies found that listeners had difficulty perceiving a higher-level organization of musical structure, especially for the completeness and coherence of large-scale tonal relationship (Gotlieb and Konecni, 1985; Cook, 1987; Karno and Konecni, 1992; Deliege et al., 1996; Tillmann and Bigand, 1996, 2004; Tillmann et al., 1998). Until recently, Koelsch et al. (2013) first explored the neural responses to the processing of nested structure and found that structurally irregular endings elicited larger early right anterior negativity (ERAN) and N5 components than structurally regular endings, reflecting the structural integration for long-distance dependence. Similar components, such as N5 and late positive component (LPC), were also observed in music nested structure processing by Chinese listeners (Ma et al., 2018a,b; Zhou et al., 2019). These results demonstrated the integration of harmonic cadence into the originally tonal context and the cognitive processing of nested structure.

Considering the important contribution of structure to emotion in music, the present study examined the tension experience induced by the nested structure and its underlying neural mechanisms. We manipulated the structural type while keeping the cadence unchanged and created three conditions as follows: non-nested structure, singly nested structure, and doubly nested structure. Frequent key changes were included in the nested structure but not in the non-nested structure, leading to different ways of prolongational reduction. A real-time tension rating task was employed, that is, the tension value was continuously recorded during the unfolding of the whole pieces to reflect the dynamic and time-varying characteristics of tension experience (Fredrickson, 2000; Hackworth and Fredrickson, 2010; Schubert, 2010). Given that the pattern of tension experience was determined by prolongational reduction, we predicted that the nested structure would induce higher tension than the non-nested structure due to frequent key modulations. In particular, the more complex the structure was, the more fluctuations would occur in the pattern of tension and resolution experience. Furthermore, given that the key point representing the tonality return was the cadence in the nested structure, the event-related potential (ERP) responses were locked to the final chords. Based on the cognitive processing of nested structure reported in previous studies (Koelsch et al., 2013; Ma et al., 2018a,b; Zhou et al., 2019), we also predicted that larger N5 or LPC would be elicited by the nested structure compared to the non-nested structure, reflecting the cognitive processing of long-distance structural integration.

## Materials and Methods

### Participants

*A priori* sample size was calculated using G*power (G*power version 3.1.9.4), and the result indicated that a sample of 27 was required in our study to reach 90% power and for detecting an effect size of *f* = 0.30, with *α* = 0.05. The effect size was based on a previous study investigating the processing of nested structure in music (Ma et al., 2018a). Therefore, we recruited 30 subjects for our experiment. Given that the processing of doubly nested structure may be difficult for nonmusicians, we recruited musicians who had received more than 8 years of formal musical training and played at least one musical instrument. Then, we randomly selected 30 musicians (*M*_age_ = 22.34 years, SD = 2.49, 20 females) to participate in the experiment. They were graduate students in music colleges and had received formal Western instrumental training, such as piano, violin, viola, and cello, for an average of 16 years (8–19 years). In music colleges, they learned many Western music theory curricula, including Western harmony, polyphony, orchestration, music form, history of Western music, etc. They were all right-handed and all of them had no history of neural impairment or psychiatric illness. The study was approved by the Institutional Review Board of the Institute of Psychology, Chinese Academy of Sciences, in accordance with the ethical principles of the Declaration of Helsinki. All the participants provided informed consent.

### Stimuli

Ten original chorale sequences including ten bars were composed in a 2/4 meter. The original sequences started with a tonic chord in C, A, or G major keys, and then developed around the key and ended with a harmonic cadence from dominant to tonic chords. The original sequences with non-nested structure unfolded in a single key and had no modulation in the middle of the sequences.

**Figure 1:**
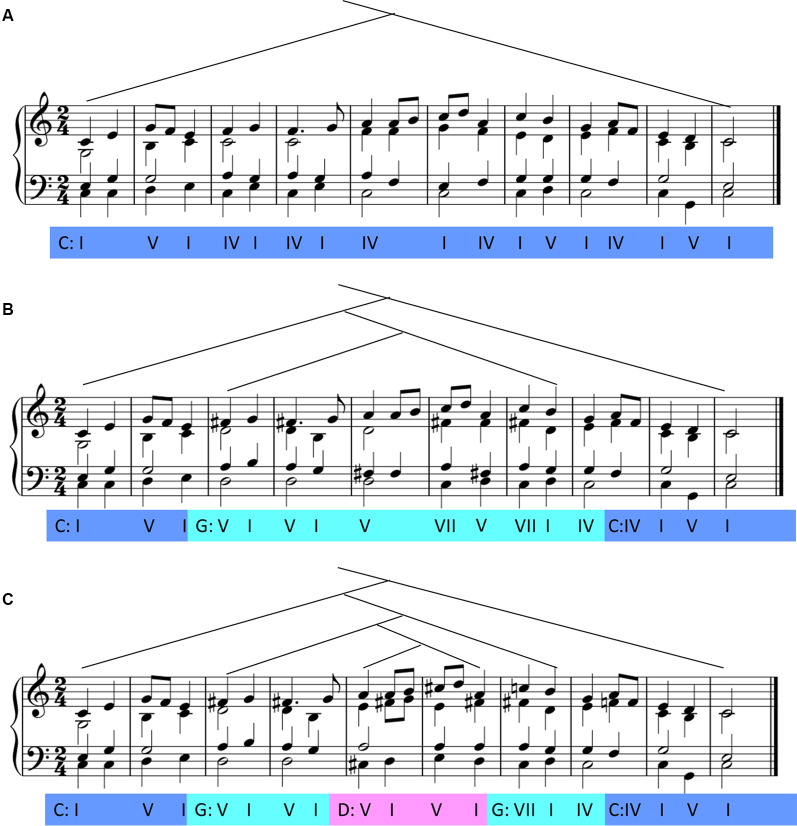
Samples of the musical sequences used in the study. Sequences with non-nested structure **(A)**, singly nested structure **(B)**, and doubly nested structure **(C)**.

The nested sequences were obtained by modulating the key in the middle of the original sequences. For the singly nested structure, the chords were not changed until the first chord in measure 3, which was the featured chord in the dominant key. The featured chord included one pitch that was in-key in the present key but out-of-key in the previous key, signifying the modulation to the dominant key. Then, the sequences developed around the dominant key until the second chord in measure 8, which was the featured chord in the initial key. For the doubly nested structure, the chords were the same as the singly nested structure except those in measures 4 and 5. The first chord in measure 4 was the featured chord in the double dominant key, following which the sequences developed around the double dominant key in these two measures. In both the singly and doubly nested conditions, the endings of the sequences were harmonic progressions from dominant to tonic chord in the initial key, signifying the modulation return into the beginning key (see Figure 1 for an example). Ten original sequences and 20 corresponding modified versions were all transposed to three other keys, yielding 120 sequences in total (10 excerpts × 4 keys × 3 structures). Using the Sibelius 7.5 software, we created the stimuli and adopted a Yamaha piano timbre with a velocity of 100 through the Cubase 5.1 software. All the stimuli files were played at a tempo of 100 beats per minute.

### Procedure

The sequences were presented in a pseudorandom order such that a given condition could not be repeated more than three times in succession and the same original sequence could not be consecutive sequences. The participants were required to judge the experienced tension continuously while listening to music, which was recorded by the Psychopy 1.0. software interface at a sampling rate of 20 Hz. They indicated the tension level by the position of a slider bar at the center of the screen, which was controlled by moving the mouse up or down. At the beginning of each trial, the slider was set to a quarter of the whole bar in order to prevent the participants’ rating out of the bar scope over the whole music piece. Three practice trials were performed before the formal experiment to familiarize the participants with the stimuli and the procedure. The stimuli were presented binaurally through Audio Technica CKR30iS headphones.

### EEG Recording and Analysis

Electroencephalography (EEG) data were recorded by Brain Products with 64 Ag/AgCI electrodes in International 10-20 system scalp locations at the sampling rate of 500 Hz. FCz was used as an online reference electrode. The electrode between Fz and FPz served as the ground electrode, and the electrode placed below the right eye was used to track eye movements. We kept the impedance of all electrodes less than 5 kΩ during the whole experiment.

The raw EEG data were preprocessed with EEGLAB (Delorme and Makeig, 2004) in MATLAB. First of all, the data were referenced to the algebraic mean of the left and the right mastoid electrodes. Second, the data were filtered offline with the Basic FIR Filter function implemented in EEGLAB to remove linear trends. We set 0.1 Hz with a filter order of 13,750 points as the lower edge and 30 Hz with a filter order of 220 points as the higher edge of the frequency pass band. Then, the data were segmented into epochs of 1,400 ms, ranging from −200 to 1,200 ms relative to the final chord. Each trial was baseline-corrected using the 200-ms prestimulus interval, and ocular and muscle artifacts were corrected using an independent component analysis algorithm (Makeig et al., 1997; Delorme and Makeig, 2004) implemented in EEGLAB. Trials in any electrode exceeding ±75 μV were regarded as artifacts and rejected. The threshold of artifact rejection was consistent with previous studies (e.g., Ellis et al., 2015; Sun et al., 2018; Zhang et al., 2018). Finally, average ERPs were calculated for each participant at each electrode in each condition.

Based on previous studies (Besson and Faïta, 1995; Patel, 1998; Regnault et al., 2001; Zendel et al., 2015) and the visual inspection, we selected 650–900 ms as the time window of LPC, the mean amplitudes of which were entered into statistical analysis. The ERPs were analyzed statistically in four regions of interest: left anterior electrodes (F1, F3, F5, FC1, and FC3), right anterior electrodes (F2, F4, F6, FC2, and FC4), left posterior electrodes (P1, P3, P5, CP1, and CP3), and right posterior electrodes (P2, P4, P6, CP2, and CP4). Repeated-measures ANOVAs taking condition (non-nested vs. singly nested vs. doubly nested), laterality (left vs. right), and anteriority (anterior vs. posterior) as within-subject factors were conducted. We conducted Mauchly’s test to test the assumption of sphericity in repeated-measures designs. If the assumption of sphericity was not met, the *p*-values corrected by the Greenhous–Geisser method were reported. Simple effect tests and planned comparisons were conducted when there were any interactions with critical manipulations in ANOVAs. Bonferroni correction was applied to adjust the multiple comparisons.

## Results

### Behavioral Results

**Figure 2:**
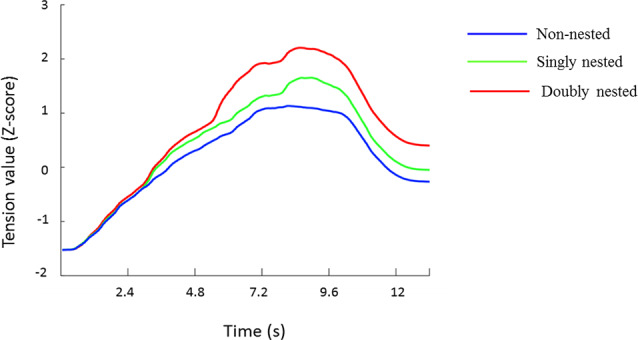
Mean ratings (Z-score) of tension values for each stimulus type at each time point. The color scheme codes represent non-nested, singly nested and doubly nested structures, respectively.

**Figure 3:**
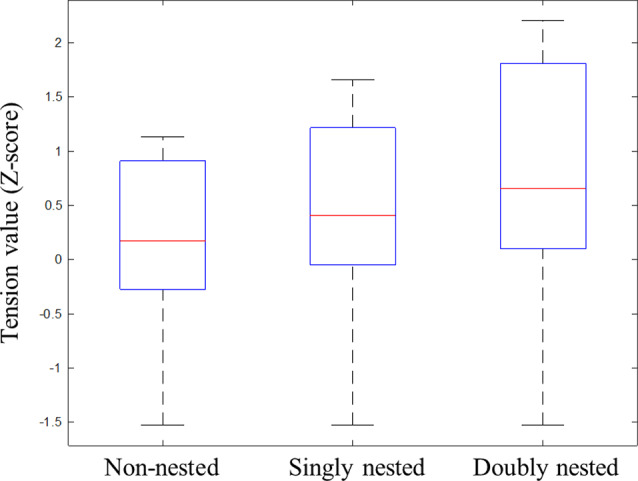
Box plots of Z-scores of tension values for the tension ratings under non-nested, singly nested and doubly nested conditions. These box plots contain the extreme of the lower whisker, the lower hinge, the median, the upper hinge, and the extreme of the upper whisker. The two hinges are the first and third quartiles, and the whiskers extend to the most extreme data.

At first, all data were normalized to Z-scores for each participant to minimize the differences across participants in terms of the slider ranges, which were also used by previous studies (e.g., Farbood, 2012; Lehne et al., 2013; Gingras et al., 2016). The tension values averaged across all participants are presented in Figure 2, showing dynamic changes in tension over the course of the whole musical sequences under the three conditions. Figure 3 exhibits the median, the first and the third quartiles, and the highest and the lowest tension values under each condition.

Given the response delays that existed in the tension rating task, we did not choose a specific time window to calculate the average tension values. Instead we first calculated the range between the highest and the lowest tension values and conducted repeated-measures ANOVA (rmANOVA) analysis with the structural types as the main factor. The results found a significant main effect of structure (*F*_(1,29)_ = 27.00, *p* < 0.001, partial *η*^2^ = 0.48). Further paired comparisons among the three conditions showed that the ranges in the doubly and the singly nested structures were larger than in the non-nested structure (doubly: *p* < 0.001; singly: *p* = 0.001), and the range in the doubly nested condition was wider than that in the singly nested condition (*p* < 0.001; non-nested: *M* = 3.00 ± 0.98; singly nested: *M* = 3.41 ± 1.00; doubly nested: *M* = 4.02 ± 1.03). Second, we analyzed the tension peaks for each subject and conducted one-way rmANOVA analysis. The results showed a significant main effect of structure (*F*_(1,29)_ = 21.24, *p* < 0.001, partial *η*^2^ = 0.42), indicating that the tension peak in the doubly and the singly nested structures was larger than in the non-nested structure (doubly: *p* < 0.001; singly: *p* = 0.005) and larger in the doubly nested structure than in the singly nested structure (*p* < 0.001; non-nested: *M* = 1.23 ± 0.97; singly nested: *M* = 1.53 ± 1.01; doubly nested: *M* = 2.08 ± 1.23).

In order to examine the process of tension induction and resolution, respectively, the difference between the original and the highest tension value (tension induction) and the difference between the highest and the final tension value (tension resolution) were calculated for each subject under each condition. In terms of tension induction, the one-way rmANOVA results showed a significant main effect of structure (*F*_(1,29)_ = 16.70, *p* < 0.001, partial *η*^2^ = 0.37), indicating that the tension difference in the doubly and the singly nested structures was larger in the non-nested structure (doubly: *p* < 0.001; singly: *p* = 0.011) and larger in the doubly nested condition than in the singly nested condition (*p* = 0.005; non-nested: *M* = 2.76 ± 1.13; singly nested: *M* = 3.07 ± 1.02; doubly nested: *M* = 3.06 ± 1.02). In terms of tension resolution, the one-way rmANOVA results also revealed a significant main effect of structure (*F*_(1,29)_ = 4.19, *p* = 0.041, partial *η*^2^ = 0.13). However, the multiple-comparisons results showed no significant difference in any paired comparisons (*p*s > 0.07; non-nested: *M* = 1.46 ± 1.01; singly nested: *M* = 1.61 ± 1.53; doubly nested: *M* = 1.72 ± 1.32).

### ERP Results

**Figure 4:**
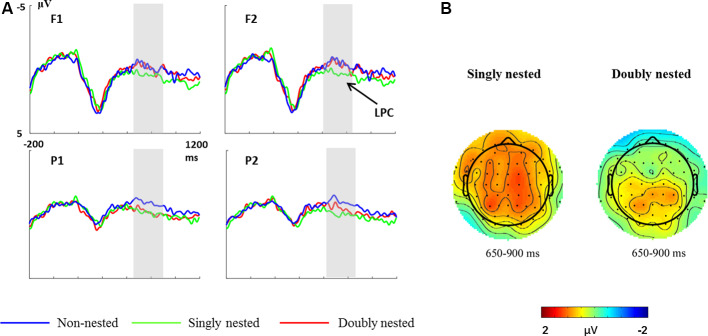
**(A)** Grand mean event-related potential (ERP) waveforms elicited by final chords in non-nested, singly nested and doubly nested structures at four electrode sites. The gray-shaded areas indicate the time window used for statistical analysis. **(B)** Scalp distributions of the singly nested minus non-nested structure difference waves and the doubly nested minus non-nested structure difference waves for the 650–900 ms latency range.

Figure 4A shows the brain electrical responses to non-nested, singly nested and doubly nested structures. Figure 4B shows the scalp distributions of the singly nested structure minus the non-nested structure and the doubly nested structure minus the non-nested structure difference waves. In the time window of 650–900 ms, the final chords in both the singly nested and the doubly nested structures elicited a larger positivity compared to the non-nested structure. However, the LPC effect elicited by the singly nested structure was distributed in the whole scalp, while the effect elicited by the doubly nested structure was only distributed in the posterior scalp.

For the time window of 650–900 ms, the one-way rmANOVA revealed an effect of structure (*F*_(1,29)_ = 5.94, *p* = 0.004, partial *η*^2^ = 0.17). Moreover, there was an interaction between structure and regions (*F*_(1,26)_ = 4.09, *p* = 0.022, partial *η*^2^ = 0.12). A further simple-effect analysis revealed that the final chords in the singly nested condition elicited a larger positivity in both the anterior (*p* = 0.048) and the posterior regions (*p* = 0.001; anterior: *M* = 0.65 ± 0.27; posterior: *M* = 0.94 ± 0.23). However, the final chords in the doubly nested condition elicited a larger positivity in the posterior regions (*p* = 0.002) than in the anterior regions (*p* = 1.00; anterior: *M* = 0.20 ± 0.29; posterior: *M* = 0.79 ± 0.20). No other significant main effect or interaction was found (all *p*s > 0.09).

## Discussion

The present study investigated musical tension induced by music sequences with nested structure and the underlying neural mechanisms, using tension experience ratings in real time and EEG recordings simultaneously. We found that the tension experience induced by the nested structure had more fluctuations than by the non-nested structure, and the difference was mainly exhibited in tension induction rather than in tension resolution. However, it was shown that a larger LPC was induced by the ending chord in nested structure compared with that in non-nested structure, and the LPC for singly nested structure had a broader scalp distribution than that for doubly nested condition, indicating that the processing of doubly nested structure was more difficult for the listeners. Following is the discussion of our main findings.

### The Whole Dynamic Tension Curves Induced by Nested Structures

The tension curves showed different tension patterns induced by nested and non-nested conditions. The tension variation range was wider for the nested conditions than for the non-nested condition because of their higher tension rising speed in the tension induction processes.

Previous studies using short chord sequences have provided evidence that tonal breaches can induce tension experience because of their violation of the established mental representations of tonal context and the prediction for the upcoming notes (Meyer, 1956; Bigand et al., 1996; Margulis, 2005; Steinbeis et al., 2006). In our study, more out-of-key chords and tonal modulations were included in the nested conditions than in the non-nested conditions, whereas rhythmic patterns and melodic contours were controlled to be consistent. Therefore, the tension increases could be more likely attributed to the frequent key modulations in the nested structures. The overlap of each tension arch associated with key modulation led to the tension increases in the sequences with nested structures. Unfortunately, we could not obtain evidence from the EEG data. In order to ensure the same final chord in each condition, the acoustic elements changed in the middle of the sequences, which hindered us from locking any specific chords to analyze ERPs in the tension induction process.

In contrast to the tension induction process, the tension curves in the resolution process were almost parallel to each other, and no significant difference was found between the tension reduction values, defined as the difference between the highest and the final tension values of each curve. Based on the assumption of prolongational reduction, all tension arches should be closed at the end of the sequences and the maximum amount of resolution should be reached (Koelsch, 2014). However, our results suggested that the listeners’ tension experience was not resolved by each key returning in a hierarchical way in the nested conditions. This could be attributed to the difficulty in perceiving and memorizing harmonic relationships in multiple nested hierarchical structures. In our study, the nested structures were shaped in the short chorale sequences with frequent key modulations. Thus, the ambiguous expectations for the tonal returning in the nested conditions might bring about very subtle variations in emotional experience and weaken the resolution experience.

From a dynamic perspective, tension resolution was slower than the process of tension induction and difficult to be resolved completely at the ending of music pieces. The reason might be that tension induction elicited by out-of-key chords was related to local violations, whereas tension resolution would be shaped by global integration. Several studies have demonstrated the difficulty of global processing in music using scrambled music pieces at different time scales and found that bar-level, but not phrase-level scrambling influenced the perception of tonal structure (Tillmann and Bigand, 1998; Granot and Jacoby, 2011). It has also been confirmed by an ERP study that the information in the local context has an earlier influence than in the global context, as reflected by an early ERAN component for local violation rather than for global violation (Zhang et al., 2018). Further study is needed to examine the difference of tension experience elicited by the processing of local and global structures.

### The Discrepancy Between Cognitive and Emotional Responses to the Final Chord

In our study, the final chords in both the singly nested and the doubly nested structures elicited larger LPCs compared with the non-nested structure. LPC is an ERP correlate of syntactic processing in language (Friederici et al., 1993; Kaan et al., 2000; Hahne, 2001; Mueller et al., 2005; Phillips et al., 2005) and music (Patel, 1998; Neuhaus, 2013; Sun et al., 2018), reflecting the integrative process and the cognitive resources allocation. Evidence of LPCs for the processing of non-adjacent tonal integration is also given by previous research on musical syntax violation (Koelsch et al., 2013; Ma et al., 2018a,b; Zhou et al., 2019). The LPC effect observed in our study may be ascribed to the more cognitive resources required by combining local information into higher global hierarchical units for the nested structure than the non-nested structure. According to Meyer (1956) and Lerdahl and Jackendoff (1983), when the music returns to the beginning tonality, the listeners would generate the feeling of harmonic completeness. This view was supported by our ERP results. Generally speaking, our results suggested that the listeners were able to process the long-distance harmonic dependency in the complex structures.

However, the behavioral data showed no significant difference in the resolution process, although the tension value induced by the last chord seems to be different between the nested and the non-nested conditions. The tension declines with a similar slope from the tension peak to the ending of the whole chorale sequences so that the difference in ending values should be ascribed to tension accumulation. Interestingly, our unpublished data (under review) also found the phenomenon that the tension experience elicited by structural violations was not resolved entirely and immediately at the ending of each phrase but accumulated during subsequent music pieces. Taken together, our studies suggested that the dynamic temporal mode in which musical tension experienced instantly was influenced by previous time windows and produced an additionally increased tension experience (Farbood, 2012).

Combining the behavioral and the EEG data together, it seems that the cognitive processing of the distant tonal relationships in the nested structure did not bring about a resolution experience. It may be explained in terms of the relationship between cognition and emotion. It has been acknowledged that activations of both the autonomic nervous system and cognitive evaluation are prerequisites for the emotional experience (Schachter, 1959, 1964). In music, cognitive evaluation is also one of the mechanisms underlying emotional induction (Juslin and Västfjäll, 2008; Juslin, 2013). Although the experience of tension and resolution relies heavily on cognitive processing, insufficient physiological activation cannot definitely elicit the experience. Our study supported the emotional theory by demonstrating the divergence of cognitive and emotional implementations.

### The Influence of Nested Complexity on Tension Experience

In our study, two types of nested structures induced different tension experiences. The tension experience was more dramatic in the doubly nested condition than in the singly nested condition, with a wider tension range and a higher tension peak in the doubly nested condition. Furthermore, the tension curves indicated an acceleration of tension rise in the doubly nested condition than in the singly nested condition, which might be attributed to the number of subcomponents inserted into the main phrase, as the occurrence of each subcomponent increased the tension experience. To our knowledge, this is the first study to reveal the difference in tension experience induced by the singly nested structure vs. the doubly nested structure in music.

Compared with the non-nested structure, the LPC elicited by the singly nested structure is distributed in the whole brain, whereas the LPC elicited by the doubly nested structure is only found in the posterior brain area. In addition, our study also suggested that the cognitive processing of the doubly nested structure was more difficult than that of the singly nested structure, given that the distribution and the amplitude of the LPC were modulated by task difficulty (Gunseli et al., 2014; Bertoli and Bodmer, 2016; Timmer et al., 2017). Consistent with our results, one previous study also found more difficult processing for the doubly nested structure than the singly nested structure while using atonal music and artificial grammars of interval and melodic lines to construct the nested structures (Cheung et al., 2018). Language materials with more nested structures required longer reading time (Babyonyshev and Gibson, 1999; Nakatani and Gibson, 2010) and activated more activities of the left pars opercularis in the case of controlling working memory load (Makuuchi et al., 2009) compared with the fewer nested structures.

In conclusion, the tension experience elicited by the nested structure was higher and had more fluctuations than that by the non-nested structure. Furthermore, the difference was mainly exhibited in tension induction rather in resolution experience. Although the explicit resolution experience was unaffected by the nested structure, larger LPCs were elicited by the ending chords in the nested condition than in the non-nested condition, reflecting the divergence between cognitive integration and the resolution experience. Given that the LPC effect elicited by the doubly nested structure has a smaller scalp distribution than the singly nested structure, we speculated that it was more difficult for listeners to integrate the final chords into such a complex musical context. Our study demonstrated the influence of nested structure on tension experience and revealed dynamic and different processes for tension induction and resolution for the first time.

Given that the processing of a doubly nested structure may be difficult for nonmusicans, only highly proficient musicians were included in our study. Although previous studies have found that both Western and Chinese nonmusicians exhibited specific neural responses to integrate tonally long-distance dependency, the musical sequences with doubly nested structure were barely used in their studies (e.g., Koelsch et al., 2013; Ma et al., 2018a,b). Future studies should investigate whether nonmusicans can process musical tension induced by complex structures and the influence of musical training on such processing. Moreover, despite the fact that tension is the basis for emotion induction in music, we know little about how musical tension contributed to the emotion experience. Thus, more attention should also be paid to the relationship between musical tension and emotion induction in real music pieces. Investigations focusing on the above issues will shed new light on the mechanisms of musical emotion processing.

## Data Availability Statement

The datasets generated for this study are available on request to the corresponding author.

## Ethics Statement

The studies involving human participants were reviewed and approved by Institute of Psychology, Chinese Academy of Sciences. The patients/participants provided their written informed consent to participate in this study.

## Author Contributions

LS and YY proposed the study and designed the experiment. LS and CF conducted the tests. All the authors contributed to data analysis, drafting and revising the article.

## Conflict of Interest

The authors declare that the research was conducted in the absence of any commercial or financial relationships that could be construed as a potential conflict of interest.
